# Rational Use of Medicine in Children—The Conflict of Interests Story. A Review

**DOI:** 10.5041/RMMJ.10371

**Published:** 2019-07-18

**Authors:** Klaus Rose, David Neubauer, Jane M. Grant-Kels

**Affiliations:** 1 klausrose Consulting, Pediatric Drug Development & More, Riehen, Switzerland; 2 Department of Child, Adolescent and Developmental Neurology, University Children’s Hospital, Ljubljana, Slovenia; 3 Department of Dermatology, UConn Health, Farmington, Connecticut, USA

**Keywords:** Developmental pharmacology, juvenile idiopathic arthritis, pediatric drug development, pediatric investigation plan (PIP), pediatric oncology

## Abstract

**Background:**

United States (US) and European Union (EU) legislation attempts to counterbalance the presumed discrimination in pediatric drug treatment and development.

**Methods:**

We analyzed the history of drug development, US/EU pediatric laws, and pediatric studies required by US/EU regulatory authorities and reviewed relevant literature.

**Results:**

The US and EU definitions of a child are defined administratively (rather than physiologically) as being aged <17 years and <18 years, respectively. However, children mature physiologically well before their seventeenth or eighteenth birthdays. The semantic blur for these differing definitions may indicate certain conflicts of interest.

**Conclusions:**

Pediatric healthcare today is better than ever. Regulatory-related requirements for “pediatric” studies focus on labeling. Most of these studies lack medical usefulness and may even harm “pediatric” patients through administration of placebo and/or substandard treatment, despite the resultant publications, networking, patent extensions, and strengthened regulatory standing. Clinicians, parents, and ethics committees should be aware of these issues. New rules are needed to determine new pharmaceutical dose estimates in prepubescent patients, and when/how to clinically confirm them. Internet-based structures to divulge this information should be established between drug developers, clinicians, and regulatory authorities. A prerequisite for the rational use of pharmaceuticals in children would be to correct the flawed concept that children are discriminated against in drug treatment and development, and to abandon separate “pediatric” drug approval processes.

## INTRODUCTION

Preconceptions and long-standing traditional treatments can be tenacious and difficult to change. There have been many incorrect opinions related to a number of pediatric conditions, including the idea that depression,^1^,^2^ schizophrenia,^3^ adult-type cancers,^4^,^5^ and more are not found in children. Today, these are recognized pediatric conditions. Prescription medications that have been United States (US) Food and Drug Administration (FDA)-approved in adults, but not in children, represent another area fraught with misconceptions.

Legislation in the USA and the European Union (EU) has sought to counterbalance the presumed discrimination in pediatric drug development and treatment.^6^,^7^ The need for such development has been stressed and endorsed by academia,^8^ regulatory authorities,^9^–^12^ and the pharmaceutical industry.^13^–^15^ The FDA and the European Medicines Agency (EMA) require pediatric studies, many of which have worldwide recruitment,^16^–^19^ paid for by pharmaceutical companies.

In 1900, few efficient systemic drugs existed, and industrial production of antibiotics only began during the Second World War. Today, drug efficacy and benefit–risk ratios are not only well documented but also undergo an extensive approval process. Pharmaceuticals have a huge impact on society, ranging from intended medical use to their societal and economic impact: prescribed by physicians, pharmacy sales, public debates, insured versus uninsured costs and reimbursement, healthcare jobs, therapeutic expectations by the public, and more. In addition, the role of regulatory authorities has transitioned from administrative organizations to powerful institutions, and clinical studies now have a major role in the drug approval process. Drugs both make lethal diseases treatable,^20^,^21^ and create great wealth for the pharmaceutical industry.

Originally “drug labels” (labeling) simply described the packaged medication. Since 1906, drug labeling has evolved to include therapeutic characteristics.^22^ Responding to the 1962 thalidomide disaster, FDA approval became based upon *pre-approval* clinical studies, a principle now accepted worldwide.^23^ The term “off-label” emerged in 1988,^24^ reflecting the FDA’s growing administrative influence. Although US legislation does not prohibit off-label use or off-label prescriptions, it does forbid interstate commerce of misbranded food and drugs.^22^ The FDA dislikes off-label use and promotion of such, and has collected billions of dollars in fines for it.^22^ However, this conflicts with the physician’s right of discretion and is hotly debated in the courts.^22^,^25^

Central regulatory authority involvement has changed and shaped society’s relationship with medications. Clinical studies are regarded as the gold standard for drug treatment decisions. Participation in clinical studies and publications have become key factors in a clinicians’ career. Many studies are sponsored by companies that anticipate retrieval of invested money via post-approval sales.

Conflicts of interest exist when professional judgment concerning a primary interest, including patient welfare or the validity of research, may be influenced by another interest. Healthcare, approval of effective drugs, and the pharmaceutical industry itself are entangled in a world where conflicts of interest abound. Beyond financial compensation, clinicians profit from participation in international studies by related international meetings, networking, conference presentations, and publications. “Evidence-based medicine” suggests that medical decisions are based on evidence.^26^,^27^ However, most studies are co-designed by clinicians and industry representatives with naturally different goals. Evidence-based medicine is lauded,^27^ relativized/ridiculed,^28^ or openly criticized.^29^ To prevent fraud, only studies listed in a publicly available registry before study initiation are considered for publication in better peer-reviewed journals.^27^

This review discusses—and thereby opens a Pandora’s box for—these issues.

## MATERIALS AND METHODS

We reviewed literature related to the history of drug development, the FDA approval process, and how children entered into this equation. We examined the ramifications of international recruitment, EU pediatric legislation, exemplary clinical areas for pediatric studies triggered by regulatory decisions, and how academia reacted and behaved. Regulatory documents were internet-retrieved.

## RESULTS

### Children as “Therapeutic Orphans”

The concept of children as “therapeutic orphans” began in 1962, when the FDA started to control prescription medicine advertising and corporate lawyers started inserting specific pediatric warnings into drug labeling.^30^–^32^ These warnings were based on reported toxicities in preterm newborns treated with antibiotics in the 1950s, and intended to mitigate lawsuits in the litigious USA.^31^,^32^ However, Shirkey, the first chairman of the American Academy of Pediatrics (AAP) committee on drugs, claimed that these warnings deprived children of modern drugs.^6^ Soon after, the AAP and FDA began to closely collaborate.^8^ In 1979, the FDA defined children as being 16 years or under (<17).^7^ Since 1997, US law rewards pediatric studies with 6-month patent extensions, for which companies must accept and execute FDA “written requests.” Furthermore, since 2003, the FDA has been authorized to mandate pediatric studies also without reward.^7^ This concept pretends that administratively defined “children” remain as immature and vulnerable as preterm newborns until they are adults.^31^–^34^

### Publications Supporting the FDA Stance

The FDA’s concept is discussed and justified in several papers, claiming that: (1) drugs prescribed for children were not sufficiently studied in children; (2) pharmaceutical companies have limited interests to study drugs in “children;” and (3) lack of pediatric studies and pediatric labeling leads to additional risks.^4^,^5^,^35^–^38^ Most publications do not discuss treatment in preterm newborns but in the “pediatric population” (<17 years). However, 15-year-old adolescents are legally underage, administratively FDA-defined as “children,” but, with regard to metabolism, are no longer children. Snyder and colleagues believed that dosing in “neonates, infants, toddlers, children, and adolescents” requires understanding of pharmacokinetics and pharmacodynamics in each age group,^4^ although the reference cited states the opposite about adolescents, who need adult dosages.^39^

The focus of pediatric FDA-requested studies has been regulatory, not clinical. A connection between pediatric labeling, improved clinical use, and avoidance of adverse events is claimed;^4^,^5^,^35^–^38^ FDA authors also claim that physicians must decide between withholding treatment proven effective in older patients, or prescribing off-label, with doses based on untested hypotheses, placing children at increased risk of adverse events.^38^ A 1977 AAP guideline states it is “unethical to adhere to a system which forces physicians to use therapeutic agents in an uncontrolled experimental situation virtually every time they prescribe for children.”^40^^(pp91–92)^ These positions would be true if children remained as vulnerable as premature newborns until their seventeenth birthday.

Clearly, the needs of very young children versus older children have been confused. The limited awareness of infant vulnerability to drugs in the 1950s/1960s has been translated into a generalized warning of alleged treatment dangers in “children,” ignoring physical maturation. The literature maintains this semantic blur for different meanings of “children,” i.e. the very young versus the FDA-defined child <17.^4^,^5^,^35^,^38^ This confusion led to FDA requests for separate pediatric efficacy and safety studies,^41^ although most are clinically and medically unjustified.

### The EU Steps In

In 2006, the EU defined “children” as being under 18 years of age (<18).^42^ Pediatric investigation plans (PIPs) were required for every new drug, unless the targeted disease was on the list of “class waivers,” i.e. conditions not found in children. The EMA has been continuously revising this list since 2008 and removed, for example, adolescent melanoma.

The EMA PIP scheme is mechanistic, often requiring placebo-controlled efficacy studies for multiple sclerosis,^43^ allergic rhinoconjunctivitis,^44^ and leukemia^45^ drugs in the “pediatric” population. Open-label studies are required on pharmacokinetics, safety, and activity for acute myelogenous leukemia (AML) and/or chronic myeloid leukemia (CML) pharmaceuticals,^45^ as well as many compounds treating acute lymphoblastic leukemia (ALL). International “pediatric” studies with centers in Switzerland,^16^ the USA and Russia,^17^ China,^18^ Germany,^46^ and Slovenia^19^ include PIP-required “pediatric” studies for drugs treating several conditions, including juvenile idiopathic arthritis and diabetes. While the FDA has relented in areas such as atopic dermatitis^47^ and epilepsy,^48^,^49^ the EMA continues to demand separate “pediatric” studies.

### Examples from Specific Clinical Areas

The confusion engendered by the administrative definitions of “child” and the bureaucracy involved for pharmaceutical approvals is evident in a number of clinical areas.

#### Depression

Use of antidepressants in pediatrics has a confusing history that leaves physicians in a quandary. Contributing to this is the administrative definition of “children,” which prevents young patients from receiving effective depression treatment. Suicide in young persons is a higher-ranked cause of death than malignomas.^50^ Suicide is often caused by depression, hence antidepressive treatment is of high clinical importance. As shown in Table 1, childhood depression was not considered a reality, although today its existence is undisputed.^51^–^53^ The FDA became involved in an attempt to reduce suicidality in children, to no avail.

**Table 1:** Changes in View and Management of Depression in Young Persons.

Date/Time Period	Stance and Management
1970s	Childhood depression considered non-existent^1^
1990s	Childhood depression undisputed^1^,^51^–^53^
Since 1997	FDA rewards 23 placebo-controlled pediatric studies to test efficacy of antidepressants in “children” The United States Treatment of Adolescents with Depression Study (TADS) is nationally funded Pediatric antidepressant efficacy studies with mixed results^1^,^54^–^59^
2004	FDA issues black-box warning for antidepressants in children, adolescents, and young adults because of their association with suicidality Studies referenced by FDA not designed to assess suicidality^58^,^59^ Decrease in prescriptions for SSRIs and antidepressants in young patients, and increased incidence of suicide^58^,^59^ Fluoxetine the only antidepressant FDA-approved in “children”^60^,^61^
2009	FDA approves escitalopram for major depressive disorder (MDD) in “children”^62^
To date	Academic researchers continue to accept the division into adult versus “pediatric” populations; they perform studies, analyses, meta-analyses, and demand more research^63^–^67^

Today, pediatricians and psychiatrists treating depression in minors must either follow regulatory recommendations or administer off-label prescriptions for potentially life-saving medications. The American Psychiatric Association (APA) stated that antidepressants save lives, with “no care at all” being the greatest threat to a depressed child. It expressed concerns that the black-box warning could reduce appropriate prescribing. The American Academy of Child and Adolescent Psychiatry (AACAP) has stated that the black-box warning was inconsistent with research and clinical experience.^54^ However, representative pediatric and psychiatric bodies have not acknowledged the suicidal consequences of the black-box warning, nor have they challenged the definition of “children.”^32^ Furthermore, the committee decision leading to the FDA’s black-box warning was not unanimous,^1^ and there was disagreement between clinicians and the FDA’s interpretation of the related studies.

#### Oncology

Pediatric oncology studies triggered by the FDA endorse the semantic blur of bureaucratically defined “children.” These studies assumed that, except for CML, the biology of malignancies differs in adults versus children.^4^,^5^ The exception for imatinib was made because it was the first personalized anticancer drug for CML and was from early on known to work in both adults and children.^68^

The FDA issued 25 written requests for carcinoma drugs; only clofarabine, everolimus, and imatinib received pediatric labels.^4^ These study requests were made despite the prior extensive (and successful) use of the investigated pharmaceuticals in different combinations of up to 13 anticancer drugs in pediatric oncology.^69^

The pemetrexed written request asked to investigate “refractory or relapsed pediatric malignancies” in infants >1 month to adolescents.^70^ A subsequent pemetrexed publication reported its tolerance in “children and adolescents with refractory solid tumors, including CNS tumors,” with no evidence of objective anti-tumor activity found in the studied childhood tumors; however, the publication failed to mention the study’s regulatory background.^71^

All but one of the FDA-triggered oncology studies were open-label with one chemotherapeutic agent.^5^ The only FDA-triggered randomized pediatric oncology study was on the addition of docetaxel to the combination of cisplatin and 5-fluorouracil for nasopharyngeal carcinoma (NPC).^4^ The academic publication described NPC as a malignancy in children and adolescents and did not reveal the study’s regulatory background.^72^ However, NPC affects children, adolescents, and adults.^73^,^74^ The study showed no difference in the treatments^72^ but led to a patent extension for the sponsor.^75^

One FDA-program rewarded pediatric melanoma studies with ipilimumab, but 13 PIPs demanded “pediatric” studies in solid tumors, including melanoma. Two PIP-demanded “pediatric” melanoma studies had to be terminated because monotherapy with ipilimumab and vemurafenib, respectively, became sub-standard and recruitment waned.^31^,^32^,^34^ Five studies are still recruiting worldwide.^31^,^32^

Fludarabine studies revealed relatively low efficacy.^76^–^79^ Labeling was not changed, but the company received a patent extension, as did the others that fulfilled FDA written requests.^75^,^80^

The FDA-required pediatric oncology studies were not designed to promote survival and quality of life, as did the earlier studies performed by the pediatric oncology researchers,^69^,^81^ but to provide regulatory coverage for compounds already used successfully by clinicians. Many academic publications describe their respective study rationale as a scientific challenge.^77^,^82^–^85^ The need for separate “pediatric” studies is taken for granted and not critically discussed. The reason why companies sponsor(ed) such studies was/is omitted.

The FDA-requested pediatric oncology studies were mostly performed in heavily pretreated, relapsed or refractory patients,^5^ raising inappropriate hopes for families and patients.^78^ Some studies resulted in labeling changes, others not. Clofarabine studies did not improve life expectancy or symptoms,^79^ but the manufacturer received a patent extension.^75^

The FDA-requested pediatric oncology studies were not scientifically motivated, in contradistinction to the pediatric oncology networks studies.^69^,^81^ Instead, they provided patent extensions for the sponsoring companies.^75^,^80^ In written requests, the FDA misleadingly stated that study participation was standard-of-care in pediatric oncology.^70^ While this was/is true for therapeutically focused studies,^69^,^81^ this is not true for regulatory FDA-triggered studies.

Finally, not all malignancies in underage patients are “pediatric” cancers—for example, conventional melanoma,^86^ or ALL, where re-programmed leukocytes destroy ALL cells.^20^ Tisagenlecleucel is FDA/EMA-approved in recurrent or refractory B-cell precursor ALL in ≤25-year-olds;^21^ this age limit reflects that young patients’ leukocytes are easier to re-program than those of older patients. Also this age limit has a certain degree of arbitrarity, but it is not justified by blurring legal and physiological terms. Furthermore, tisagenlecleucel is not a “pediatric” drug for a “pediatric” disease; it is a drug that works in relatively young patients.

#### Hypertension

Hypertension, frequent in adults, is rare in younger patients. The FDA rewarded “pediatric” antihypertensive studies,^87^ although hypertension did not reflect a serious gap in pediatric healthcare.^88^ The patients ranged from 6 to 16 years^87^ and were recruited by administrative age limits. However, beta-blockers work equally in 18- or 15-year-olds; hence, effective treatment was and is denied to adolescents who could profit from them.

#### Diabetes

Four oral drugs for type 2 diabetes mellitus (T2DM) have been FDA-evaluated for “pediatric” use: metformin, glimepiride, rosiglitazone, and metformin+ glyburide. Each drug reduced glycemic parameters; three failed to reach the FDA-demanded efficacy threshold, and only metformin was FDA-approved in “children.” These studies did not contribute to better diabetes treatment in children. The academic publication described “pediatric drug development” as what it is: regulatory approval/non-approval of drugs whose efficacy in humans is already well proven.^89^

#### Neonatology and Infectious Diseases

Neonatology has continuously advanced.^90^ Neonatal studies are demanded by representatives of “pediatric drug development.”^8^,^36^ In very-low-birthweight (VLBW) neonates, antifungals are already used clinically, not with a regulatory focus, but with a focus on patients’ wellbeing.^91^

Chloramphenicole toxicity in neonates is well known. The toxicities in preterm newborns that triggered industry’s pediatric warnings occurred with antibiotics.^33^ Among the current pediatric clinical challenges in antifungal treatment are prophylaxis and treatment of invasive fungal infections (IFIs) in premature and VLBW neonates, candidemia, and meningoencephalitis in neonates, and prophylaxis, empiric therapy, and targeted antifungal therapy in children with immunodeficiencies.^91^ The PIPs for the antifungals posaconazole, voriconazole, and isavuconazonium demand regulatory efficacy confirmation of these compounds in underage patients. Antifungal prevention and treatment is done today, without separate approval in VLBW neonates. Hence, there is no medical sense in demanding separate proof of efficacy of antifungals for young patients.

#### Multiple Sclerosis

Lacking scientific rationale are the FDA and EMA requirements for active-controlled or even placebo-controlled comparisons of anti-inflammatory compounds in multiple sclerosis (MS).^43^ While the clinical course of pediatric versus adult MS is different, the disease itself is inflammatory.^92^ The FDA/EMA assume that drugs not separately approved might not work before a minor turns 17 or 18.

#### Pediatric Clinical Pharmacology

Pediatric clinical pharmacology had and has a key role in promoting separate “pediatric” studies.^8^ In Europe, many publications claimed that off-label drug use resulted in higher rates of adverse events.^93^–^95^ However, the major statistically significant finding of Turner et al. was the number of medications administered to patients, not their unlicensed or off-label status.^94^ In another study, the data “suggest an increasing risk of adverse drug reactions related to off-label drug use,” but the authors emphasize that this risk would be acceptable should further studies confirm the potential benefit of such drug use.^95^ The claim that off-label use “doubles the frequency of adverse drug reactions”^93^ was not and is not based on data.

### Demands to Expand Current Pediatric Legislation

The multi-stakeholder group “ACCELERATE” (www.accelerate-platform.eu/) discusses pediatric oncology studies. Without acknowledging the flaws of US/EU pediatric legislation, it recommends inclusion of adolescents in promising adult cancer studies,^96^ a suggestion also recommended by Geoerger et al. following the terminated “pediatric” ipilimumab study.^97^

Pediatric researchers and regulatory/industry representatives propose switching from organ-specific PIPs to a “mode of action” approach,^98^–^100^ without acknowledging the flaws of US/EU pediatric laws. The “mode of action” approach would lead to more “pediatric” studies with modern anti-cancer compounds. The tisagenlecleucel PIP EMEA-001654-PIP01-14-M02 demands separate “pediatric” studies despite its approval in young patients.

The newest EMA class waiver changes^101^ will lead to “pediatric” hepatic carcinoma studies. Comparable to conventional melanoma, hepatic carcinoma rarely occurs in patients under 18 years of age. Separate “pediatric” studies based on artificial age limits are questionable.

### Political Stage and Further Plans

The 2016 FDA report^102^ and a resolution of the European Parliament^103^ ask for an expansion of pediatric legislation. The US “Research to Accelerate Cures and Equity” (RACE) for Children Act will come into force in 2020.^104^–^106^. It will remove current restrictions that exclude orphan drugs from FDA-mandated pediatric trials, expanding the FDA’s authority to demand “pediatric” cancer studies. It is endorsed by *Nature* journal and more than 100 advocacy organizations.^107^ It will strengthen the FDA’s administrative power and trigger more “pediatric” studies, but will not advance pediatric cancer treatment.

## DISCUSSION

In those rare diseases for which pediatric therapy is different than in adults, e.g. bisphosphonates for osteogenesis imperfecta,^48^,^49^ or anastrozole for McCune–Albright syndrome,^108^ separate pediatric efficacy studies make sense. However, for most diseases occurring in adults and children, once medication efficacy is established, adolescents can usually be treated with adult doses. Dose-finding is necessary only for prepubescent children, and, clearly, neonates need specific attention. Hence, pharmaceutical companies should include adolescents in pivotal studies.

In some areas, the FDA has become less dogmatic: for partial onset seizures (POS) epilepsy, efficacy extrapolation is accepted from adults down to 4-year-olds;^48^ for topical treatment of atopic dermatitis pivotal studies have been accepted with patients aged 2–79 years.^47^

As stated above, “pediatric drug development” originated as a response to the US thalidomide catastrophe. New procedures were imposed on the administratively defined pediatric population in an attempt to keep dangerous substances from the market and improve pediatric healthcare; the main proponent were the AAP and the FDA, endorsed by the clinical community. For many practitioners common sense prevailed, as noted already by Shirkey in 1968: most clinicians ignored the pediatric warnings.^6^ The flawed US approach was further augmented by the EU. With development of more efficacious treatments, subjecting young patients to traditional “standard of care” or placebo is more likely to result in substandard treatment. Young patients suffering from lethal conditions can actually be directly harmed by being placed in comparator or placebo groups.

The key issue is the administrative definition of the “pediatric” population: <17/<18.^7^,^31^,^32^ “Pediatric drug development” requirements are based on a semantic blur of different physiological, administrative, and legal meanings of the word “child.” The “moral imperative”^33^ for “pediatric” studies appeals to protective instincts toward young children. Endorsed by the clinical community, the translation of this concept into law and the bestowal of executive power to the FDA created incentives for questionable, expensive, and harmful “pediatric” studies. The EU followed, augmented, and expanded on the US precedent. The mandatory US law “Pediatric Research Equity Act” (PREA)^7^ does not apply to orphan designations; EU PIPs are also required for rare diseases, vaccines, and biologics. The stronger mandate for the EMA reflected back on the FDA which now asks for “initial Pediatric Study Plans” (iPSPs); at least the iPSP template^109^ is less demanding than its PIP counterpart.^110^

The flawed definition of “children”—supplemented by FDA assumptions about pediatric cancer^4^ and juvenile “suicidality”^58^,^59^—has confused the clinical world. Procedurally, “pediatric” studies are well documented, but they are based on a flawed concept. There were and are pockets of resistance,^28^,^88^,^111^,^112^ but open intellectual challenges of FDA/EMA pediatric activism are still rare.^16^–^19^,^31^,^32^,^43^–^46^ The EMA’s claim that it made more medicines “available” for children^10^ is misleading. “Available” means EMA-issued pediatric labels: a regulatory, not a clinical achievement.

## CONCLUSIONS

Many publications on “pediatric” studies pretend to investigate a scientific question and omit mentioning that the studies were FDA/EMA-required.^77^,^82^–^85^,^89^ Others discuss “pediatric drug development,” but the discussed studies are only regulatory in nature.^8^

Ongoing research is required in neonates. Institutional review boards/ethics committees should re-assess all ongoing pediatric studies. Those found to be questionable should be suspended and newly submitted questionable ones rejected.

Based on this review, we believe that (1) treatment of neonates, infants, children, adolescents, and adults is today better than ever in history; (2) the justifications for separate “pediatric drug development” are flawed; (3) these flaws are not a conspiracy, but reflect the complex path toward rational use of drugs, biologics, devices, and technology; (4) the flawed concept of “pediatric drug development” has created many conflicts of interest since becoming US law; (5) this flawed US-born concept was adopted and augmented by the EU; (6) the EU further potentiated conflicts of interest of many parties and institutions that profit from separate “pediatric” studies; (7) the EU exaggerations finally facilitated detection of the fundamental flaws; and (8) academic critical reflections have not pinpointed the flaws of “pediatric drug development.”^88^,^112^

It is possible that underlying conflicts of interest will trigger angry responses from individuals, parties, and institutions; the ensuing public debate could rock public trust in science and institutions. Nevertheless, conflicts of interest are not only financial and do not follow traditional boundaries of institutions; hence, protective mechanisms against fraud and professional misconduct are needed and should be reflected in required revisions to US/EU pediatric legislation. Also, the International Committee of Medical Journals Editors (ICMJE) guidelines should be accordingly revised.^27^ These steps will eventually facilitate better access to efficient drugs, biologics, devices, diagnostics, and breakthroughs in young patients.

The current framework allowed and allows pediatric researchers on the one hand to support pediatric legislation and demand more pediatric studies, but on the other hand to omit, in the resulting study publications, the regulatory background and the reason companies had to sponsor the studies.^87^ In the future, regulatory demands that trigger “pediatric” studies should be clearly mentioned in academic publications. The ICMJE guidelines should be accordingly revised.^27^ Today, clinicians have to choose between prescribing effective treatment off-label, or prescribing substandard treatment on-label. Dose-finding in prepubescent children is necessary, but not separate drug approval.

The FDA- and EMA-triggered “pediatric” studies might represent the largest abuse of patients in medical research, dwarfing even the atrocities unveiled by Beecher in 1966 and other projects that led to the Belmont Report.^113^–^115^

New guidelines are needed, including when and how drug developers should estimate doses in prepubescent patients and when and how to confirm them in “opportunistic” settings.^116^,^117^ Internet-based information structures for dose recommendations of new drugs in prepubescent patients will technically be rather easy to establish in collaboration with drug developers, clinicians, and regulatory authorities.

Finally, a prerequisite to moving ahead is requiring the FDA, EMA, AAP and its European counterparts to reject the flawed concept of children as “therapeutic orphans” and the need for separate drug approval in “children.” This flawed concept is now outdated and should be discarded.

## Abbreviations

AACAP: American Academy of Child and Adolescent Psychiatry
AAP: American Academy of Pediatrics
ALL: acute lymphoblastic leukemia
APA: American Psychiatric Association
CML: chronic myeloid leukemia
EMA: European Medicines Agency
EU: European Union
FDA: United States Food and Drug Administration
IFIs: invasive fungal infections
iPSP: initial pediatric study plan
ICMJE: International Committee of Medical Journal Editors
MDD: major depressive disorder
POS: partial onset seizures
PIP: pediatric investigation plan
PREA: Pediatric Research Equity Act
RACE: Research to Accelerate Cures and Equity (RACE) for Children Act
SSRIs: selective serotonin re-uptake inhibitors
T2DM: type 2 diabetes mellitus
US: United States of America
VLBW: very low birthweight.
